# Supplementary material for: Ultrafast one-chip optical receiver with functional metasurface
Source: Nat Commun. 2025 Nov 24;16:10070. doi: 10.1038/s41467-025-65984-6 (PMC12644511; doi:10.1038/s41467-025-65984-6)
Supplement: Supplementary file 1 — Supplementary Information [file 41467_2025_65984_MOESM1_ESM.pdf]

Supplementary Information for

Ultrafast one-chip optical receiver with functional metasurface

Go Soma,[1,*] Tomohiro Akazawa,[1] Eisaku Kato,[1] Kento Komatsu,[1]
Mitsuru Takenaka,[1] Yoshiaki Nakano,[1] and Takuo Tanemura[1,†]
[1]*School of Engineering, The University of Tokyo, Tokyo, Japan*

Supplementary Note 1. FULL-WAVE SIMULATIONS OF METASURFACES

To rigorously evaluate the performance of the designed metasurfaces (MSs), we performed full-wave simulations using the finite-difference time-domain (FDTD) method (Ansys Lumerical).

Metalens

Figure S4a shows the simulation model for the metalens (ML) presented in Fig. 2 of the main text. For computational efficiency, a scaled-down ML was simulated with half of the original design parameters ($f_1 = 410$ µm, $f_2 = 262.5$ µm, diameter ~ 180 µm), while all other parameters were kept identical to the fabricated device. The simulation domain was 185 µm × 185 µm × 5.05 µm, bounded by perfectly matched layers (PML).

A Gaussian beam with a waist of $w_{\mathrm{fiber}} = 10.4$ µm at the fiber facet was used as input. The output field after the ML was monitored and propagated to the photodetector (PD) plane via the angular spectrum method (ASM) [1]. The focusing efficiency was defined as the ratio of the optical power collected within the PD aperture to the input Gaussian beam power.

Figure S4b shows the simulated electric field distribution after the ML at 1550 nm, exhibiting the expected phase profile. Figure S4c presents the electromagnetic power distribution at the PD plane, confirming that the field is well confined within the 6-µm PD aperture. The spectral dependence of the focusing efficiency is plotted in Fig. S4d, reaching $\sim 85\%$ at 1550 nm and remaining above 70% over 1500–1600 nm.

Metasurface for Stokes-vector receiver

We next evaluated the performance of the MS for the Stokes-vector receiver (SVR) by half-scale simulations. Figure S8a shows the calculated field intensity profiles at the PD plane for input polarization states P_1–P_4 (top; see Fig. 4a in the main text) and their orthogonal states P_1'–P_4' (bottom). The MS separates and focuses each polarization state onto the designated PD.

Figure S8b presents the focusing efficiencies at the four PDs for the different polarization inputs. The average focusing efficiency, defined as the total power collected by the PDs relative to the input power, is 74%. The extinction ratio, defined as the ratio between the detected power at PD_n for the intended polarization state P_n and that for the orthogonal state P_n', exceeds 14 dB. The sensitivity penalty L—defined as the SNR degradation in the polarization retrieval process arising from imperfect splitting and optical loss (see Ref. [2])—is calculated to be 1.74 dB.

Metasurface for coherent receiver

Finally, we simulated the MS used in the coherent receiver (CR) at the original scale. Figure S11a shows the calculated intensity distributions at the PD plane for input polarizations of $\pm 45^\circ$ linear (a/b) and right/left-handed circular (RHC/LHC; r/l) states at 1550 nm. Each state is clearly focused onto the designated PD positions. Residual unfocused light arises from the interleaving of two independent MSs (MS-A and MS-B), which slightly distorts the lens aperture for each polarization [3].

Figure S11b summarizes the simulated transmission spectra at the four PD ports. The total focusing efficiency exceeds 87% across the C-band, with extinction ratios greater than 20 dB for all input states.

* go.soma@tlab.t.u-tokyo.ac.jp
† takuo.tanemura@tlab.t.u-tokyo.ac.jp

Supplementary Note 2. CROSSTALK FOR MULTI-CHANNEL IM-DD RECEIVER

We experimentally evaluated the crosstalk between channels in the four-channel receiver (Fig. 3 in the main text). Figure S6 shows the measured IV curves of the photodetector (PD) in channel 1 under three different conditions: (1) when the optical inputs at all four cores of the multi-core fiber (MCF) were turned ON (red), (2) when only the non-corresponding cores (i.e., the other channels) were ON (blue), and (3) when all optical inputs were turned OFF, corresponding to the dark current (black).

The results indicate that the photocurrent induced by adjacent channels (blue curve) is largely suppressed, corresponding to a crosstalk level below -25 dB.

Supplementary Note 3. COMPARISON WITH WAVEGUIDE-BASED RECEIVERS

To highlight the advantages of our approach, we compare our MS-integrated receiver with state-of-the-art waveguide-based receivers using various photonic integrated circuit (PIC) platforms with respect to the following aspects:

- **Power consumption:** Since our receiver provides equivalent optical functionality, the same digital signal processing (DSP) architecture can be used to retrieve the IQ signals. Thus, the DSP-related power consumption is expected to be comparable to existing PIC-based receivers. On the other hand, the surface-normal configuration of our receiver is advantageous in minimizing optical coupling loss, which is critical for enhancing receiver sensitivity and minimizing the energy per bit.

- **Footprint:** Table S1 compares our platform and a typical silicon photonic platform in terms of device footprints. Since our platform does not require edge couplers with spot-size converters (SSCs), which typically range from a few hundred micrometers to a few millimeters in length [4], the total footprint can be smaller. In addition, as demonstrated in the four-channel IM-DD receiver (Fig. 3 in the main text) and Ref. [5], our platform exhibits superior spatial scalability due to its surface-normal configuration, enabling the dense 2D integration of PD arrays and direct coupling from MCFs without the need for SSCs or external bulky fan-out devices.

- **Cost:** Although we have employed in-house fabrication facilities to fabricate InGaAs-membrane devices, such heterogeneous integration platforms have been undergoing rapid commoditization and are in the process of being adopted in silicon photonic processes. Additionally, high-speed Ge PDs can also be used to enable full CMOS compatibility. With these technologies, we believe our devices can be fabricated at a comparable or lower cost than conventional PIC-based receivers, due to their significantly smaller device footprint and reduced packaging costs.

- **Excess loss:** The measured excess loss of our receivers was in the range from 6 to 10 dB, which can be attributed to two main factors: ~ 5 dB originating from the PD (reflection and metal absorption), and the remainder from the MS and fiber alignment. These losses are not intrinsic and can be reduced in practice. First, the PD loss can be decreased by applying an anti-reflection coating and increasing the absorption layer thickness. In addition, rigorous numerical simulation suggests that the MS loss can be as small as ~ 1 dB, which should be achieved by optimizing the fabrication process. Therefore, the total excess loss could realistically be reduced to below 3 dB—a challenging target for PIC platforms.

Table S1. Comparison of device footprints between this work and typical Si photonic devices. SSC: spot-size converter for an edge coupler. PBS: polarization beam splitter. MMI: multi-mode interferometer.

**Receiver type**	**This work**	**Si photonics**	
	Lateral size (MS diameter)	Lateral size	Components
Single-channel IM-DD receiver	340 µm	∼ 400 µm	SSC + PD
Four-channel IM-DD receiver	260 µm	∼ 500 µm	4 × SSC + 4 × PD (+ External fanout)
SVR	260 µm	∼ 1 mm [6]	SSC + PBS + 2 × BS + 2×4 MMI + 6 × PD
CR	120 µm	∼ 1 mm [7]	SSC + 2×4 MMI + 4 × PD

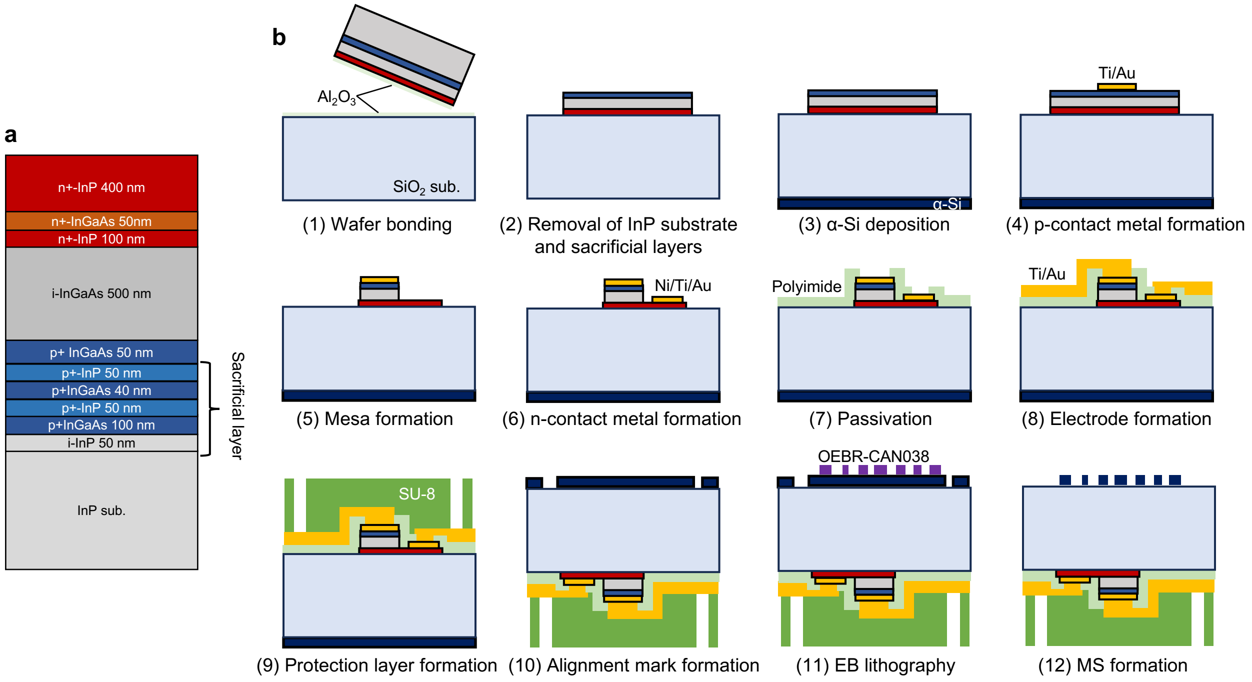

Fig. S1. Device fabrication. a, Epitaxial layers grown on an InP substrate. The InP substrate and the sacrificial InP/InGaAs layers were wet-etched after the wafer bonding process. **b**, Fabrication flow chart.

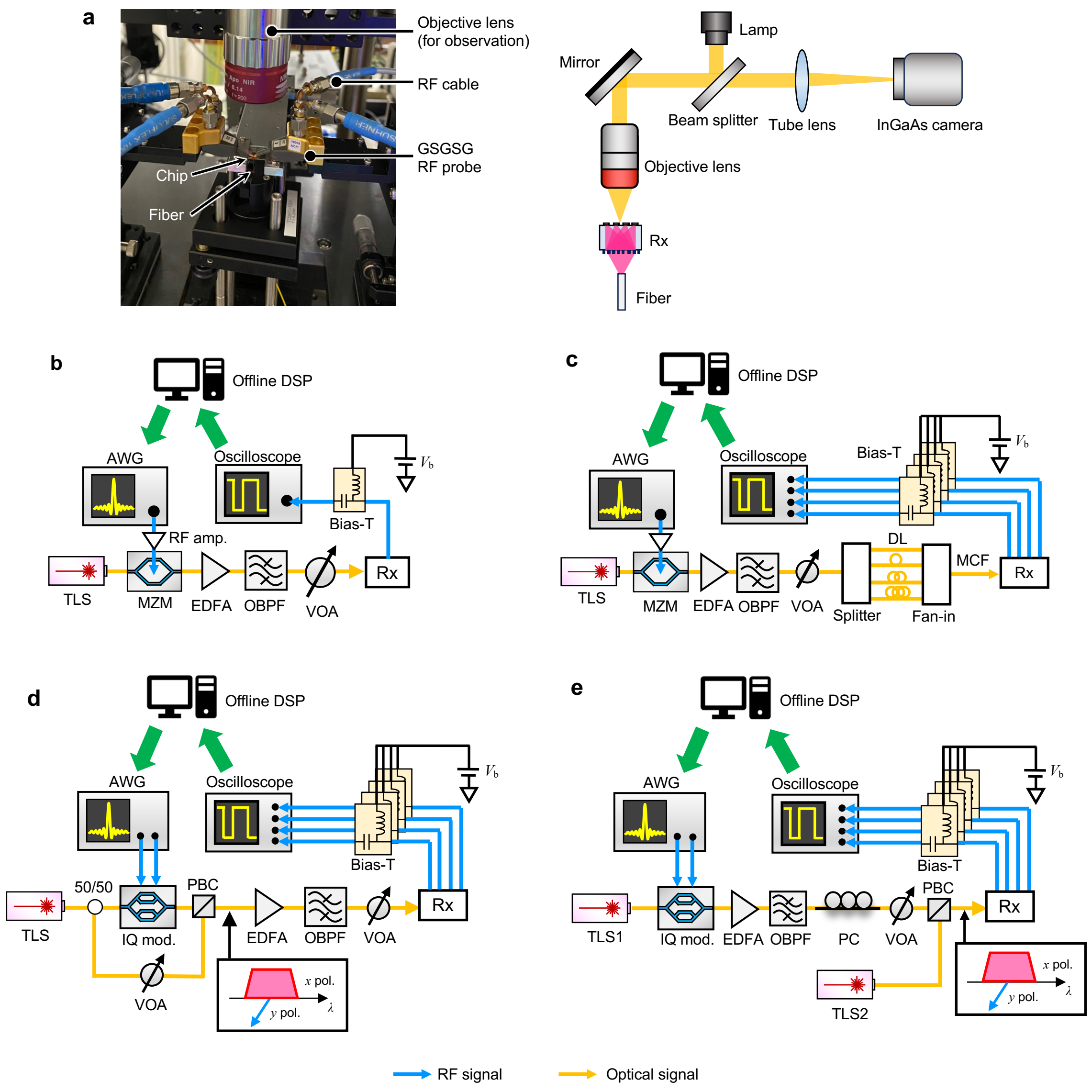

Fig. S2. Experimental setups. **a**, Photograph of the basic measurement setup (left). The two GSGSG RF probes were used to collect up to four high-speed signals. The right panel shows the schematic of the setup used to observe the positions of the chip, probes, and incident beam. **b**-e, Schematics of the setups for the high-speed signal detection experiments for (**b**) the single-channel IM-DD receiver, (**c**) four-channel IM-DD receiver, (**d**) self-coherent detection using the SVR, and (**e**) coherent detection using the CR.

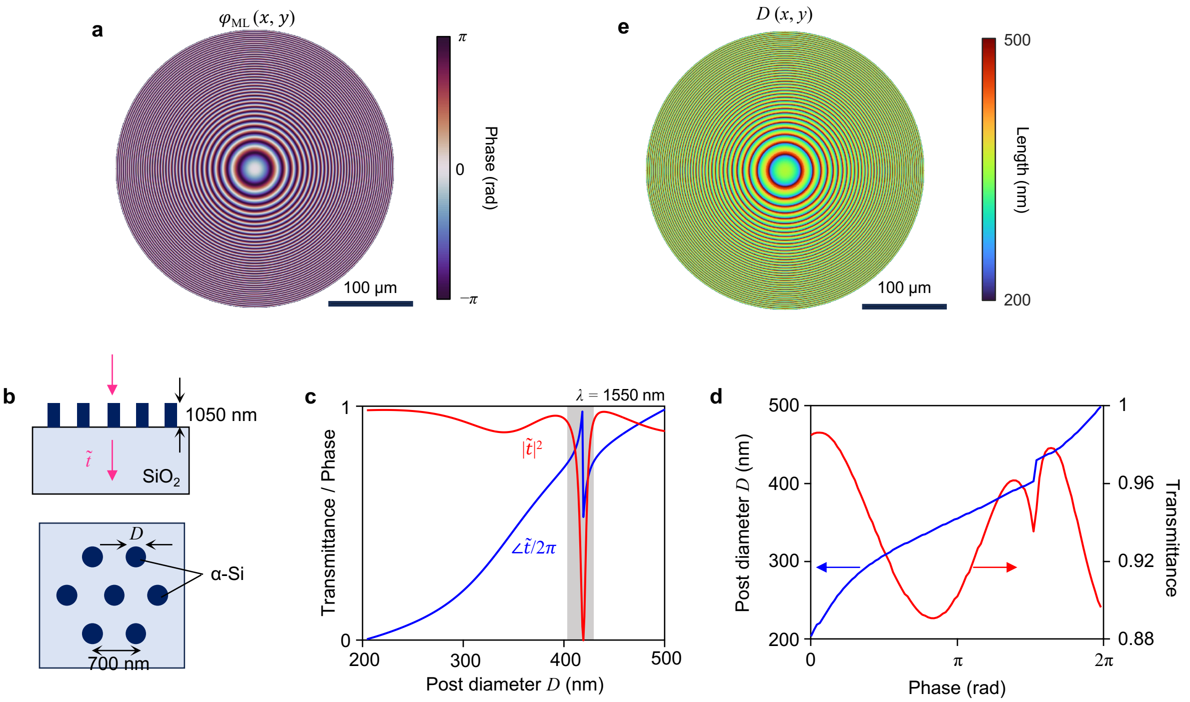

Fig. S3. ML design for the IM-DD receiver. **a**, Required phase profile $\varphi_{\mathrm{ML}}(x, y)$ to realize the ML of the IM-DD receiver in Fig. 2. **b**, Schematic of a periodic array of circular Si nanoposts arranged on a triangular lattice with a lattice constant of 700 nm and $\theta = 0$. **c**, Simulated transmittance $|\tilde{t}|^2$ and phase $\angle\tilde{t}/2\pi$ for a periodic Si nanopost array on a triangular lattice with a lattice constant of 700 nm (inset). The shaded region is excluded in the ML design to maintain high transmission. **d**, Required post diameter D to realize the required transmission phase. **e**, Spatial distribution of the designed post diameter $D(x, y)$.

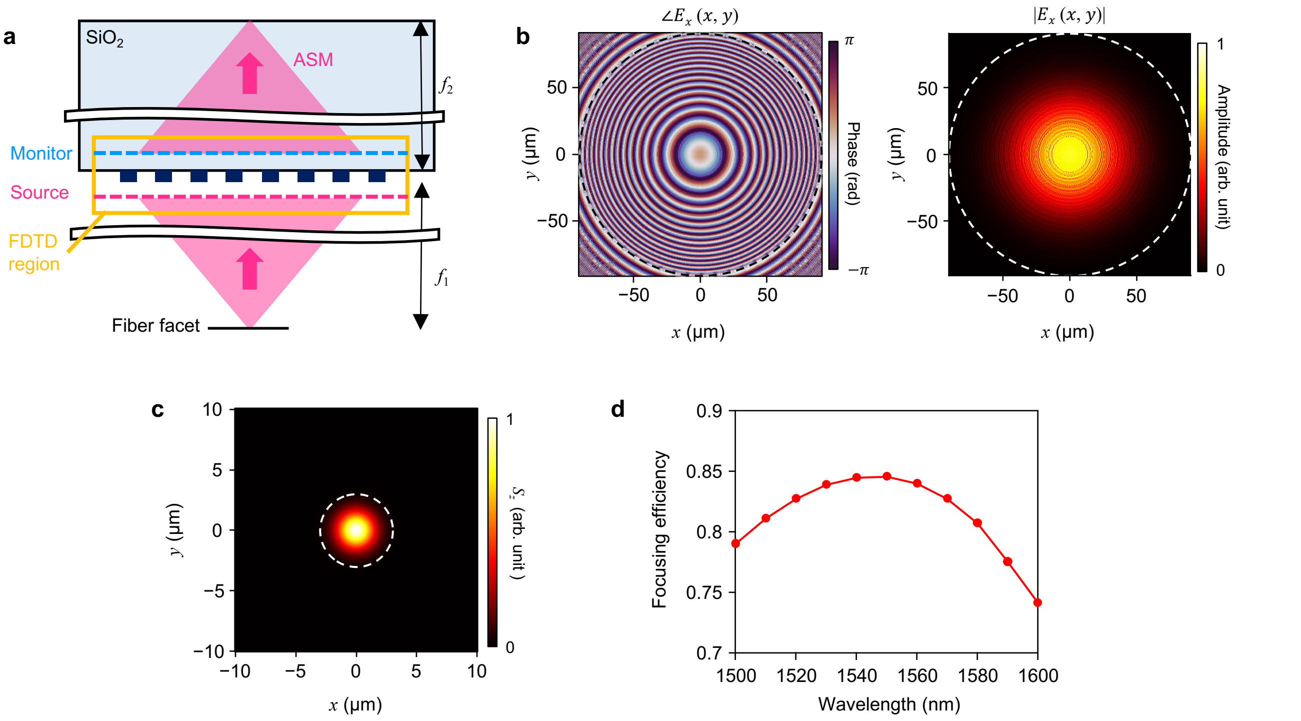

Fig. S4. Full-wave simulation of the designed ML. a, FDTD simulation model. **b**, Simulated electric field distribution after the ML at 1550 nm. The dashed line indicates the ML aperture. **c**, Electromagnetic power distribution (S_z) at the PD plane at 1550 nm. The dashed line indicates the 6-μm PD aperture. **d**, Calculated focusing efficiency as a function of wavelength.

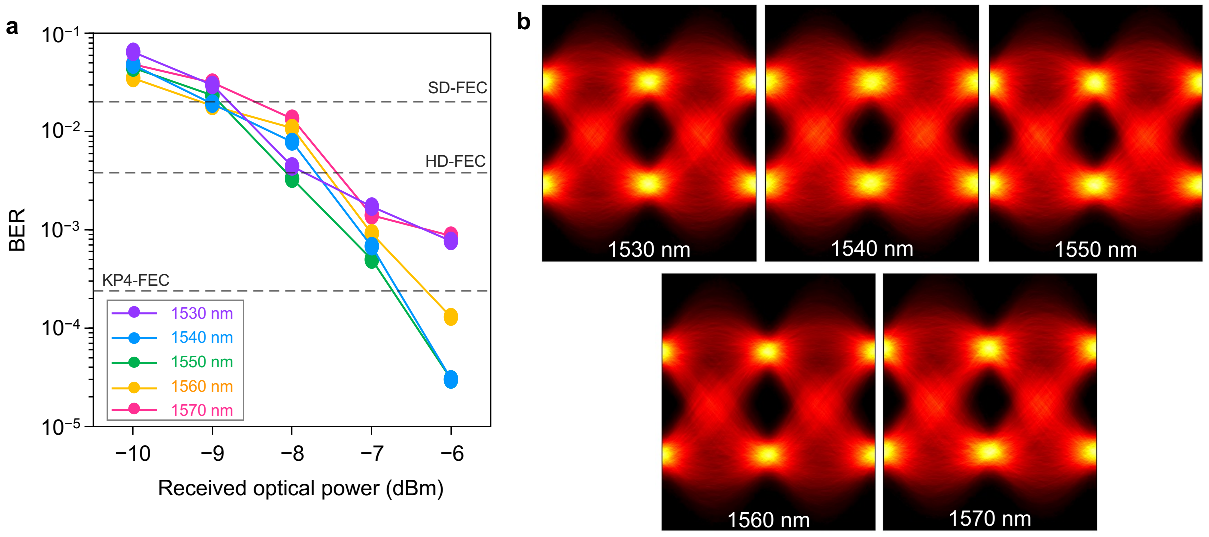

Fig. S5. Wavelength-independent characteristics of ML-integrated IM-DD receiver across the C **band. a**, Measured BER of a 40-Gbaud NRZ signal as a function of the received optical power at different wavelengths. **b**, Retrieved eye diagrams for all wavelengths.

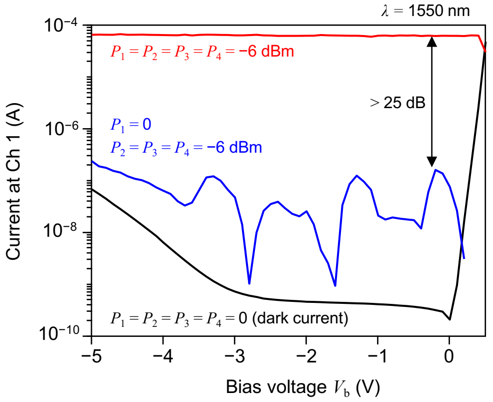

Fig. S6. Characterization of crosstalk in the multi-channel receiver. IV curves of the PD at channel 1 measured under three illumination conditions at 1550-nm wavelength: (1) all channels on, (2) only adjacent channels on, and (3) dark current.

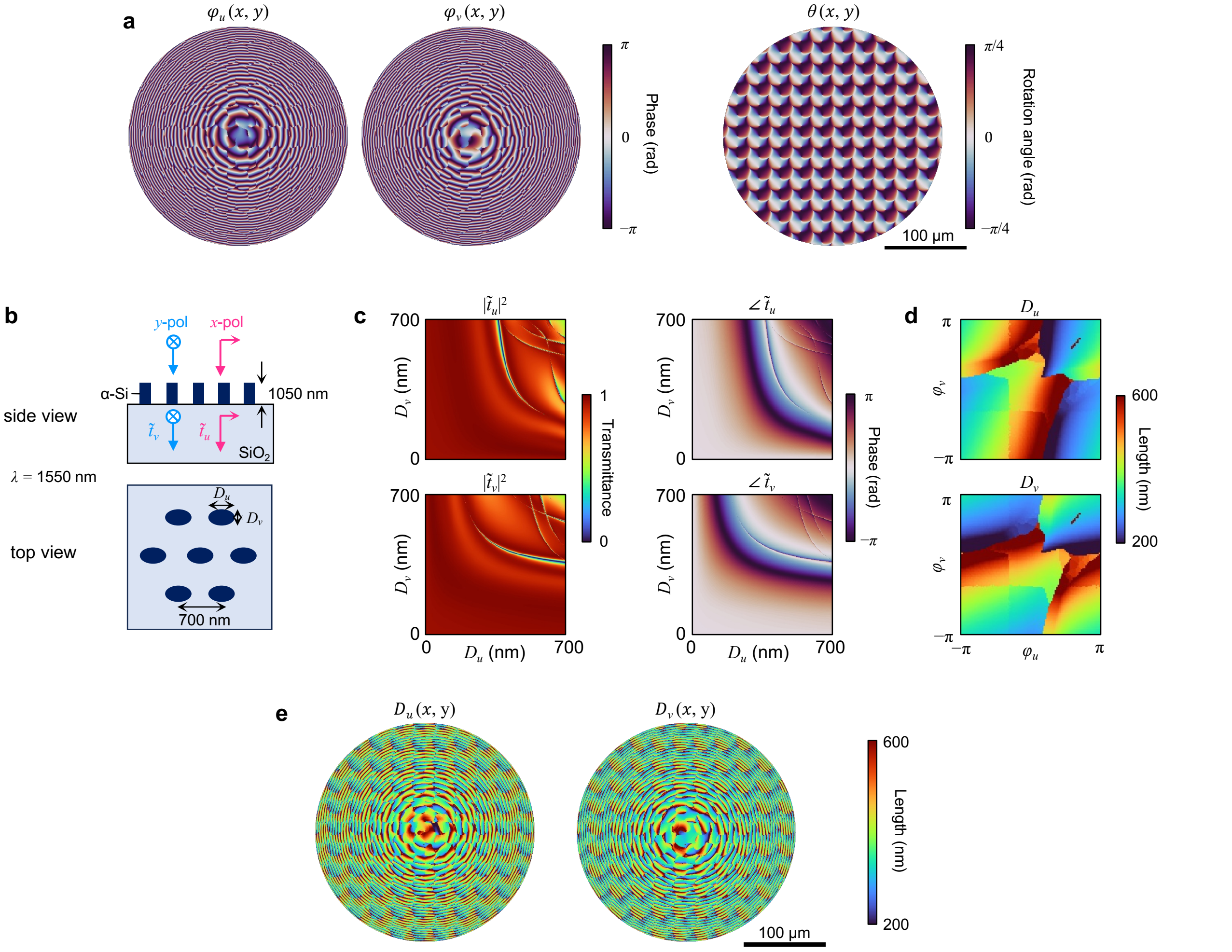

Fig. S7. MS design for the SVR. **a**, Spatial distributions of the optical parameters $\varphi_u(x, y)$, $\varphi_v(x, y)$, and $\theta(x, y)$ for the SVR described in Fig. 4 of the main text. **b**, Schematic of a periodic array of elliptical Si nanoposts arranged on a triangular lattice with a lattice constant of 700 nm and $\theta = 0$. **c**, Simulated transmission coefficients for x- and y-polarized inputs as functions of meta-atom dimensions (D_u, D_v). **d**, Optimal dimensions (D_u, D_v) of elliptical Si nanoposts that provide phase shifts of (φ_u, φ_v) to the x and y-polarized transmitted light. The dimensions were selected from 200 to 600 nm for ease of fabrication. **e**, Spatial distributions of the designed meta-atom dimensions $D_u(x, y)$ and $D_v(x, y)$.

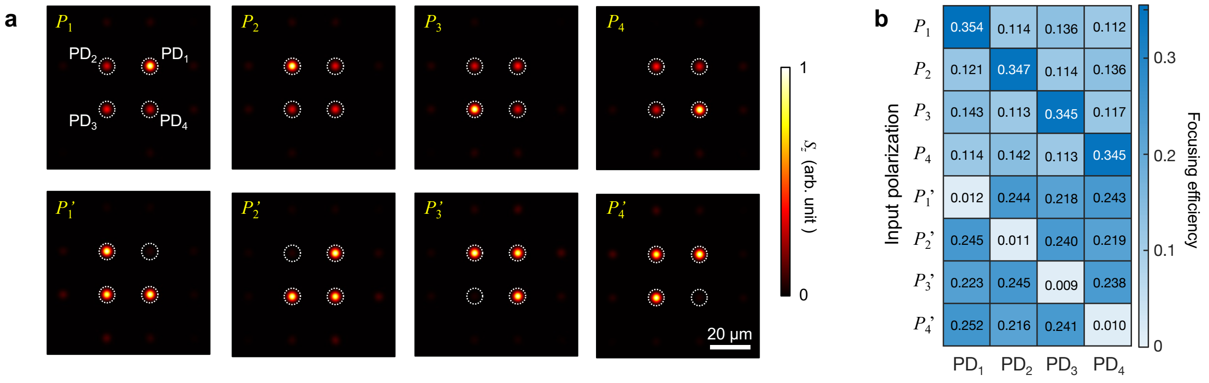

Fig. S8. Full-wave FDTD simulation of the MS for SVR at 1550-nm wavelength. **a**, Simulated intensity distributions at the PD plane for input polarization states P_1–P_4 (top) and their orthogonal states P_1'–P_4' (bottom). The dashed lines indicate PD apertures. **b**, Simulated focusing efficiencies for each PD and input polarization state.

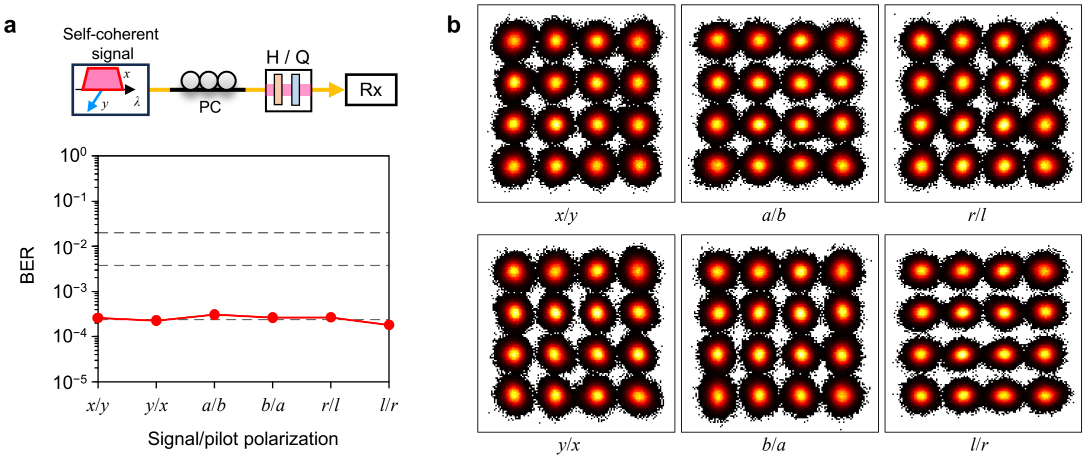

Fig. S9. Polarization-drift-resilient self-coherent detection using the SVR. **a**, Measured BER of 160-Gbit/s 16QAM signal at 1550-nm wavelength with different polarization states. Received optical power is $P_{\text{in}} = -10$ dBm. The top panel shows the schematic of the measurement setup, where we emulate arbitrary polarization drift during the transmission by rotating the HWP (H) and QWP (Q). **b**, Retrieved constellation diagrams for different input states of polarization.

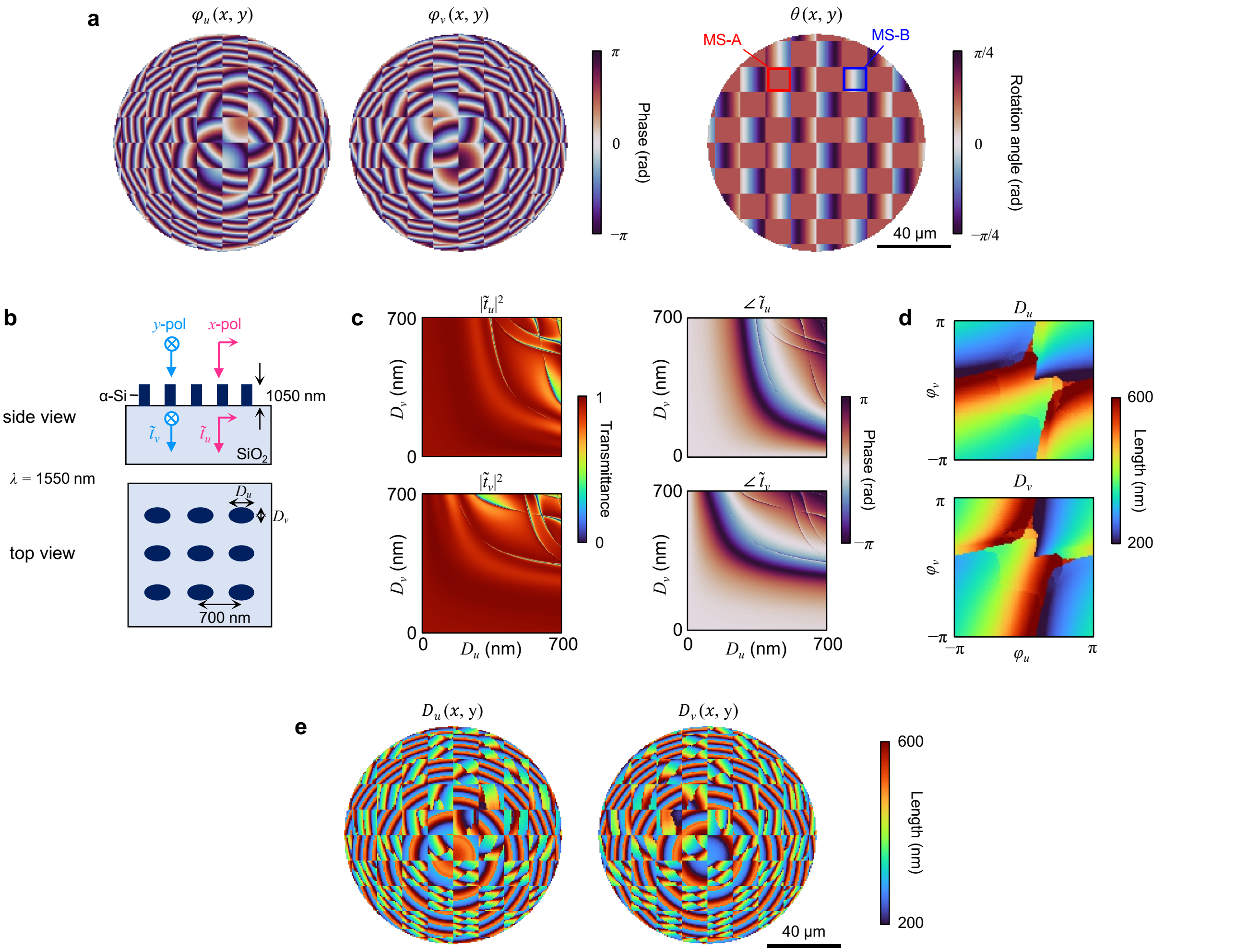

Fig. S10. MS design for the CR. **a**, Spatial distributions of the optical parameters $\varphi_u(x, y)$, $\varphi_v(x, y)$, and $\theta(x, y)$ for the CR described in Fig. 5. **b**, Schematic of a periodic array of elliptical Si nanoposts arranged on a square lattice with a lattice constant of 700 nm and $\theta = 0$. **c**, Simulated transmission coefficients for x- and y-polarized inputs as functions of meta-atom dimensions (D_u, D_v). **d**, Calculated dimensions (D_u, D_v) of elliptical Si nanoposts that provide phase shifts of (φ_u, φ_v) to the x and y-polarized transmitted light. The dimensions were selected from 200 to 600 nm for ease of fabrication. **e**, Spatial distributions of the designed post dimensions $D_u(x, y)$ and $D_v(x, y)$.

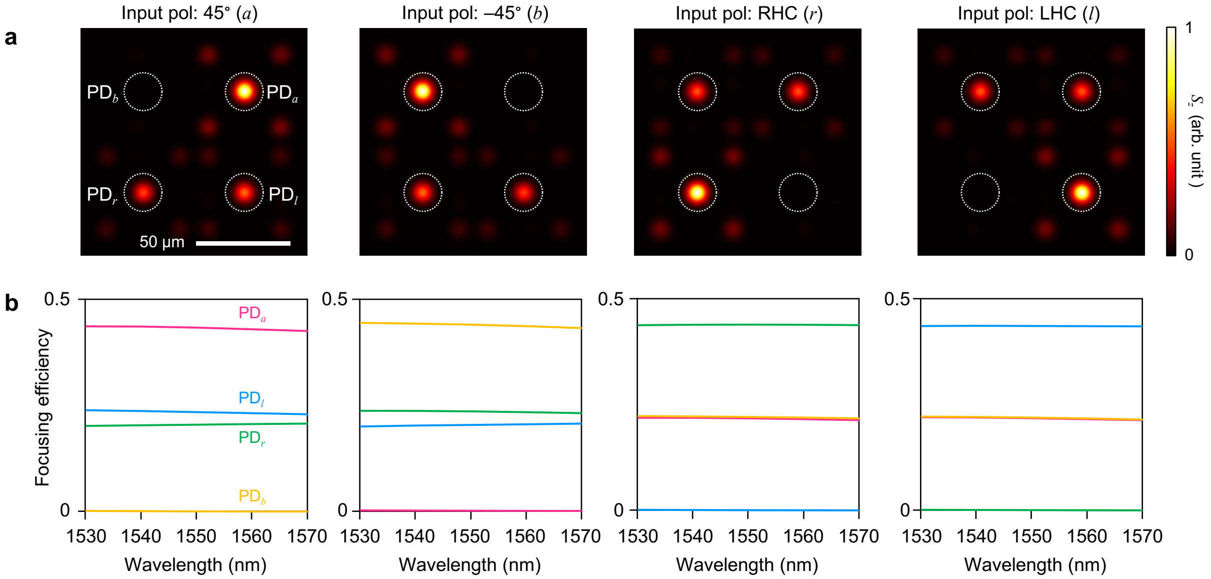

Fig. S11. Full-wave FDTD simulation of the MS for CR. a, Simulated intensity distributions at the PD plane at 1550-nm wavelength for ±45° linear (a/b) and right/left-handed circular (RHC/LHC; r/l) polarization states. **b**, Calculated focusing efficiencies at the four PD ports as a function of wavelength for different input polarizations.

[1] K. Matsushima and T. Shimobaba, Band-limited angular spectrum method for numerical simulation of free-space propagation in far and near fields, Opt. Express **17**, 19662 (2009).
[2] G. Soma, K. Komatsu, C. Ren, Y. Nakano, and T. Tanemura, Metasurface-enabled non-orthogonal four-output polarization splitter for non-redundant full-stokes imaging, Opt. Express **32**, 34207 (2024).
[3] E. Arbabi, A. Arbabi, S. M. Kamali, Y. Horie, and A. Faraon, Multiwavelength metasurfaces through spatial multiplexing, Sci. Rep. **6**, 32803 (2016).
[4] X. Mu, S. Wu, L. Cheng, and H. Y. Fu, Edge couplers in silicon photonic integrated circuits: A review, Appl. Sci. (Basel) **10**, 1538 (2020).
[5] K. Komatsu, G. Soma, S. Ishimura, H. Takahashi, T. Tsuritani, M. Suzuki, Y. Nakano, and T. Tanemura, Scalable multi-core dual-polarization coherent receiver using a metasurface optical hybrid, J. Lightwave Technol. **42**, 4013 (2024).
[6] P. Dong, X. Chen, K. Kim, S. Chandrasekhar, Y.-K. Chen, and J. H. Sinsky, 128-Gb/s 100-km transmission with direct detection using silicon photonic Stokes vector receiver and I/Q modulator, Opt. Express **24**, 14208 (2016).
[7] P. Dong, X. Liu, S. Chandrasekhar, L. L. Buhl, R. Aroca, and Y. K. Chen, Monolithic silicon photonic integrated circuits for compact 100^{+}Gb/s coherent optical receivers and transmitters, IEEE J. Sel. Top. Quantum Electron. **20**, 6100108 (2014).
